# Proteomic Profiling of *Mycobacterium tuberculosis* Identifies Nutrient-starvation-responsive Toxin–antitoxin Systems*[Fn FN2]

**DOI:** 10.1074/mcp.M112.018846

**Published:** 2013-01-23

**Authors:** Jakob Albrethsen, Jeppe Agner, Sander R. Piersma, Peter Højrup, Thang V. Pham, Karin Weldingh, Connie R. Jimenez, Peter Andersen, Ida Rosenkrands

**Affiliations:** From the ‡Department of Infectious Disease Immunology, Statens Serum Institut, Copenhagen, Denmark;; §OncoProteomics Laboratory, Department of Medical Oncology, VU University Medical Center, Amsterdam, The Netherlands;; ¶Department of Biochemistry and Molecular Biology, University of Southern Denmark, Odense, Denmark

## Abstract

In order to successfully enter the latent stage, *Mycobacterium tuberculosis* must adapt to conditions such as nutrient limitation and hypoxia. *In vitro* models that mimic latent infection are valuable tools for describing the changes in metabolism that occur when the bacterium exists in a non-growing form. We used two complementary proteomic approaches, label-free LC-MS/MS analysis and two-dimensional difference gel electrophoresis, to determine the proteome profile of extracellular proteins from *M. tuberculosis* cultured under nutrient starvation. Through the label-free LC-MS/MS analysis of fractionated samples, 1176 proteins were identified from culture filtrates of log phase and nutrient-starved cultures, and the protein levels of 230 proteins were increased in nutrient-starved culture filtrates, whereas those of 208 proteins were decreased. By means of Gene Ontology clustering analysis, significant differences in the overall metabolism during nutrient starvation were detected. Notably, members of the toxin–antitoxin systems were present in larger quantities in nutrient-starved cultures, supporting a role for these global modules as *M. tuberculosis* switches its metabolism into dormancy. Decreased abundance of proteins involved in amino acid and protein synthesis was apparent, as well as changes in the lipid metabolism. Further analysis of the dataset identified increased abundance of lipoproteins and decreased abundance of ESAT-6 family proteins. Results from the two-dimensional difference gel electrophoresis proteomics demonstrated overall agreement with the LC-MS/MS data and added complementary insights about protein degradation and modification.

*Mycobacterium tuberculosis* is a very successful pathogen that has the ability to persist in humans for decades without causing symptoms. Latent tuberculosis infection can reactivate at any point later in life and progress into active, clinically apparent tuberculosis disease.

*M. tuberculosis* differs from many other pathogens in its ability to survive in an intracellular habitat for years. It achieves this long-term intracellular persistence by controlling phagosomal maturation, preventing phagosomal fusion with the lysosome, and reducing acidification of the phagosome. To restrict *M. tuberculosis* growth, infected monocytes attract additional monocytes, macrophages, and T cells, and a granuloma is formed. Mimicking conditions thought to reflect the environment inside the granuloma *in vitro* and evaluating the transcriptional response has been the subject of intensive research in recent years. *In vitro* models have included hypoxia and nutrient starvation and have demonstrated the ability of *M. tuberculosis* to enter a stage of long-term non-replicating persistence. That this stage of the bacteria also exists *in vivo* was recently emphasized in a study of clinical sputum samples in which a persister-like *M. tuberculosis* population was identified and analyzed via transcriptome analysis (1).

Here, we took advantage of a nutrient-starvation model originating from work by Loebel *et al.* (2) and further characterized by Betts *et al.* (3). In this model, *M. tuberculosis* is resistant to isoniazid and rifampicin, as well as metronidazole, a drug effective under anaerobic conditions (3). We focused on the secretome of *M. tuberculosis*, as extracellular proteins play an important role in the host–pathogen interactions of *M. tuberculosis* and furthermore constitute a source of T cell antigens involved in a protective immune response against *M. tuberculosis* (4) and with potential as diagnostic markers (5).

In mycobacteria, several protein secretion systems have been described (reviewed in Ref. 6). The general secretion pathway (Sec) recognizes an N-terminal signal sequence in unfolded proteins, and upon export the signal sequence is cleaved off, as, for example, is observed for the lipoproteins. The twin-arginine transporter system translocates folded proteins across the cell membrane after recognition of a specific signal sequence. In addition, a novel secretion system, the type VII secretion system, responsible for the export of ESAT-6 family proteins, has recently been characterized in detail.

The recent developments in proteomics provide a unique opportunity for studying tuberculosis pathogenesis and latency. In many cases the amount of protein cannot be extrapolated from the mRNA level (7), and the localization, post-translational modification, and preferred binding partners of proteins can be revealed only through analysis at the proteomic level.

In this study, we used label-free LC-MS/MS and two-dimensional difference gel electrophoresis (DIGE)^1^ to investigate the culture filtrate proteome of *M. tuberculosis* H37Rv bacteria in normal log-phase growth and after 6 weeks of nutrient starvation.

## EXPERIMENTAL PROCEDURES

### 

#### 

##### Mycobacterial Cultures

Cultures were initiated from frozen seed stocks of *M. tuberculosis* H37Rv (ATCC 27294). For starter cultures, bacteria were grown to an optical density of 580 nm of ca. 1.0 (late log phase) in modified Sauton medium (8) under shaking conditions at 37 °C. 500-ml polycarbonate Erlenmeyer flasks (Corning, Acton, MA) containing 200 ml of modified Sauton medium were inoculated with 2 × 10^6^ bacteria per milliliter from the starter culture and placed in a standard shaking incubator at 37°C. After 7 days of growth to log phase, cultures were pelleted, washed twice with PBS, and resuspended in 200 ml PBS; this was followed by incubation for 6 weeks without shaking. Control log phase cultures were obtained after 7 days of culturing in 200 ml modified Sauton medium in 500-ml flasks at 37 °C under shaking conditions. The viability of the cultures was assessed by counting colony-forming units (cfu) of serial dilutions of triplicate cultures on 7H11 agar plates. Student's *t* test was applied to compare log_10_ transformed cfu counts at the onset and after 6 weeks of starvation.

##### Culture Filtrate Preparation and Analyses

After harvesting of cultures, the culture medium was collected, sterile filtered, and concentrated approximately 160 times in Centriprep-3 ultrafiltration units (Millipore, Carrigtwohill, Ireland). Protein concentrations were determined via the 2D-Quant method (GE Healthcare, Uppsala, Sweden).

The extracellular DNA concentration in culture filtrates (CFs) was determined using the PicoGreen^®^ double-stranded DNA Quantitation Kit per the manufacturer's instructions (Invitrogen, Abingdon, UK).

For Western blot analysis, 10%–20% Tris-Glycine PAGE^®^ precast gels (Cambrex, Rockland, ME) were used, and proteins were transferred to nitrocellulose as described elsewhere (9). The following specific antibodies were used: mouse monoclonal antibody (Mab) anti-EsxA HYB 76–8, rabbit polyclonal antibody anti-EsxB K8493, mouse Mab anti-EsxH/EsxR PV-2, and mouse Mab anti-GroEL2 HAT5. The development was performed using ECL (GE Healthcare).

##### One-dimensional Gel Electrophoresis and LC-MS/MS

The CF proteome was analyzed via gel LC-MS/MS as described elsewhere (10). In brief, the CF proteins were separated via SDS-PAGE using pre-cast 4%–12% gradient gels (NuPAGE MES system, Invitrogen) and the gels were stained with Coomassie R-250. The six SDS-PAGE lanes containing three biological replicates of log phase and starvation CF samples were cut into 10 bands, and the 60 resulting bands were processed by means of in-gel trypsin digestion according to the method of Shevchenko *et al.* (11). The volume of the gel-extracted peptide digests was reduced to 50 μl in a vacuum centrifuge. Next, the peptides were separated by means of reverse-phase chromatography (Ultimate 3000 nanoLC system, Dionex LC-Packings, Amsterdam, The Netherlands) and eluting peptides were ionized (Nanomate Triversa Chip-based nanospray source with Triversa LC coupler, Advion, Ithaca, NJ) and analyzed on an LTQ-FT hybrid mass spectrometer (Thermo Fisher, Bremen, Germany). Intact peptide masses were measured in the ICR cell and, in parallel, following a Fourier transform pre-scan, the top five peptide signals (charge states of 2+ and higher) were submitted to MS/MS in the linear ion trap.

LTQ-FT raw files were searched with Sequest using the Proteome Discoverer 1.3.0.339 data analysis package (Thermo Fisher, San Jose, CA). The following spectrum filters were applied: minimum precursor mass of 350 Da, maximum precursor mass of 5000 Da, minimum peak count of 1, and minimum S/N threshold of 1.5. The following parameters were used in the Sequest search: full tryptic specificity, maximum of two missed cleavages, maximum number of peptides considered = 500, maximum peptide output of 10, Absolute Xcorr threshold of 0, fragment ion cut-off percentage of 0.01, protein relevance threshold of 1.5, peptide relevance factor of 0.4, precursor mass tolerance of 10 ppm, fragment mass tolerance of 1 Da, and only b and y ions considered. A maximum of four modifications were allowed per peptide, and both cysteine carbamidomethylation and methionine oxidation were allowed as variable modifications. MS/MS spectra were searched against the *M. tuberculosis* H37Rv FASTA file downloaded from the Sanger Centre, with 3993 entries (created January 2010 based on GenBank accession number AL123456). Spectra were searched using Sequest, and Proteome Discoverer MSF files were imported in Scaffold 3.4.9 for peptide validation and data organization. Sequest search files were combined per biological sample (10 gel bands per sample). Peptide identifications were accepted if they could be established at greater than 95.0% probability as specified by the Peptide Prophet algorithm, which is part of Scaffold. Peptide Prophet calculates the identification probability for each peptide identification using Bayesian statistics (12). Protein identifications were accepted if they could be established at greater than 99.0% probability and contained at least two identified peptides in at least one biological sample. Protein probabilities were assigned by the Protein Prophet algorithm (13); this algorithm performed the protein inference from identified peptides and is also part of Scaffold. Proteins that contained similar peptides and could not be differentiated based on MS/MS analysis alone were grouped (designated by “+1” in supplemental Table S1). 1372 proteins were identified with a minimum of two peptides in at least one of the samples; 10 out of these 1372 identified proteins represented reversed hits, indicating a protein false discovery rate of 0.7%. The total number of forward identifications therefore was 1362 (supplemental Table S1). The mass spectrometry proteomics data (raw data, search, and peak list files) have been deposited at the ProteomeXchange Consortium with the dataset identifier PXD000111.

Relative protein quantitation was performed via spectral counting of the number of MS/MS spectra associated with an identified protein (number of “assigned spectra” as reported by Scaffold). Modified peptides were included in the spectral counts. Peptides shared between proteins (or between different protein groups) were excluded from the spectral counts. Spectral counting was used to quantify the relative abundance of individual proteins in different biological samples; spectral counting was not used for the estimation of the (relative or absolute) protein abundance of different proteins. Spectral counts were summed for each protein for each biological sample (10 gel bands). For each biological sample, the protein loading on gel, as well as the gel band pattern and intensity, was highly similar. For quantitation by means of spectral counting, lower scoring peptide matches of identified proteins also were retained. Spectral counts per protein per sample were normalized on the sum of the spectral counts for each sample (10 gel bands combined) and multiplied with the average sum across biological samples.

Significantly changed proteins were identified using the beta binomial test (14), a fold change of 1.5 (normalized spectral count ratio starvation CF/log phase) and *p* value of 0.01 were used as combined thresholds to define biologically changed proteins, and those proteins identified in starvation CF or log phase only were assigned fold changes of 100 or 0.01, respectively.

##### Gene Ontology Clustering Analysis

In order to characterize the set of increased and decreased proteins for biological interpretation, a gene ontology (GO) analysis was undertaken. Only *M. tuberculosis* proteins identified either in all three log phase CF samples or in all three starvation CF samples were included in the analysis. The protein Rv numbers were obtained from TubercuList and were used for defining the list of total detected proteins in the dataset. GO terms for identified proteins were extracted from UniProt, and overrepresented functional categories for differentially abundant proteins were determined using the GOMiner tool (15). A cutoff *p* value of less than 0.05 was used, and for overlapping GO groups, one representative category was selected within the dataset. The minimum size of the categories was set at five proteins.

##### Two-dimensional DIGE Analysis

Each gel experiment included the log phase and starvation CF samples in triplicate. Two-dimensional DIGE was performed as described elsewhere (16). Briefly, 50 μg of each sample was prepared for two-dimensional DIGE using the 2D Clean-up kit (GE Healthcare) and resolubilized in 30 mm Tris, 7 m urea, 2 m thio-urea, and 4% CHAPS, pH 8.5. Cy2, Cy3, and Cy5 minimal labeling was performed with 125 pmol of each CyDye according to the manufacturer's instructions (GE Healthcare), and isoelectric focusing with pH 4–7 immobilized pH gradient strips (GE Healthcare) was performed as described elsewhere (17). Cy2-, Cy3-, and Cy5-labeled samples were applied during the rehydration step in 8 m urea, 2% CHAPS, 0.5% immobilized pH gradient buffer, and 18 mm DTT. The second-dimension separation was performed in 10% to 20% Tris-glycine SDS-PAGE 16 cm × 16 cm gradient gels in the Protean IIxi system (Bio-Rad, Richmond, CA). Each two-dimensional gel included a log phase and a starvation CF sample, as well as a Cy2-labeled internal standard (a pool of all samples in the experiment) to allow the correction of inter-gel variation. Labeling was performed so that both log phase and starvation CF samples were labeled with Cy3 and Cy5 to account for the preferential labeling of proteins by either of the CyDyes. After electrophoresis, the gels were scanned by a Typhoon 9410 gel imager, and spot images were analyzed with the Image Master Platinum 2.0 software (GE Healthcare). The Cy2-labeled standard was used for normalization by the default normalization algorithm; spots that displayed a more than 1.5-fold difference in volume ratio, *p* < 0.05 (Student's *t* test), were selected for further characterization, and the fold change was calculated for each spot. The proteins identified in starvation CF or log phase only were assigned fold changes of 100 or 0.01, respectively. The two-dimensional DIGE gels were then silver stained using the method developed by Blum *et al.* (17), adapted for mass spectrometry compatibility as described elsewhere (18), except that methanol was replaced by ethanol. Spots selected for identification were then excised manually and digested with trypsin.

##### Protein Identification by Peptide Mass Fingerprinting and MS/MS

Peptides were analyzed via MALDI-TOF-MS and MALDI-TOF-MS/MS on an ABI 4700 TOF-TOF or ABI 4800 TOF-TOF instrument (Applied Biosystems, Foster City, CA). External calibration was performed using a tryptic peptide mixture of bovine beta-lactoglobulin. Mass spectra were analyzed using the software package MoverZ (*m*/*z*) (version 2001.08.08; Genomic Solutions, Ann Arbor, MI); the peak lists were internally calibrated, and keratin and trypsin contaminants were removed using the PeakErazor program (version 2.02, Lighthouse Data, Odense, Denmark). Peak lists were searched against the NCBI database (20091218, 10227800 sequences or 20100728, 11505486 sequences) using Mascot (version 2.2.06, Matrix Science, London, UK) in the peptide mass fingerprinting search or the MS/MS ion search. Fixed modifications included the carbamidomethylation of Cys, and variable modifications included the oxidation of Met. The peptide mass tolerance was set at ±25 ppm, the maximum number of missed cleavages by trypsin was 1, and all entries were included in the search.

## RESULTS AND DISCUSSION

### 

#### 

##### The Nutrient-starvation Model

The nutrient-starvation model employed in this study was based on work published by Loebel *et al.* and Betts *et al.* (2, 3, 19) and was used in order to compare the proteome profile of *M. tuberculosis* log phase cultures in 7H9 Middlebrook medium with that of cultures left standing in PBS for 6 weeks. To allow comparison between CFs from log phase cultures grown in nutrient-rich medium and those from nutrient-starved cultures in PBS, we replaced the 7H9 Middlebrook medium with the Sauton medium, which contains no protein addition such as albumin-dextrose-catalase enrichment. *M. tuberculosis* was cultured for 7 days in Sauton medium under shaking conditions; the bacteria were then washed with PBS and resuspended in PBS in sealed bottles for 6 weeks without shaking. Comparison of the log_10_ cfu ± S.D. at the onset of starvation (6.17 ± 0.18) with that of 6-week-starved cultures (5.90 ± 0.10) showed no significant difference in cfu values, which supports the conclusion that the bacteria enter a non-replicating state during starvation, in agreement with previous observations (3). CFs were harvested from starved and log phase cultures, sterile filtered, and concentrated for proteome analysis.

##### Global Findings of LC-MS/MS Analysis

For in-depth proteomics analysis via LC-MS/MS, the proteins in the three log phase and three starvation CF samples were fractionated via SDS-PAGE (supplemental Fig. S1), and this was followed by in-gel digestion and analysis of the resulting tryptic peptide mixtures. In total, 12,399 unique peptides derived from 1362 proteins were identified in the six CF samples (supplemental Table S1). For subsequent analyses, 1176 proteins were selected that were detected in either all three log phase samples (906 proteins) or all three 6-week-starved CF samples (1017 proteins) (supplemental Table S2).

A quantitative comparison using spectral counting as a measure of protein abundance and applying the beta-binominal test showed that 438 proteins displayed significantly different abundances (>1.5-fold difference, *p* < 0.01) when the log phase and starved CF protein levels were compared (supplemental Table S3); 230 CF proteins displayed significantly greater abundance relative to log phase CF, and 208 proteins displayed significantly lower abundance (supplemental Table S3). The double-stranded DNA content was determined in CF samples by means of the Picogreen DNA assay, and the DNA concentrations were at the same level in log phase and starvation CF (data not shown); therefore, considerable lysis of the starved cultures is not a likely explanation of the different protein compositions of six-week-starved CFs.

**Fig. 1.**
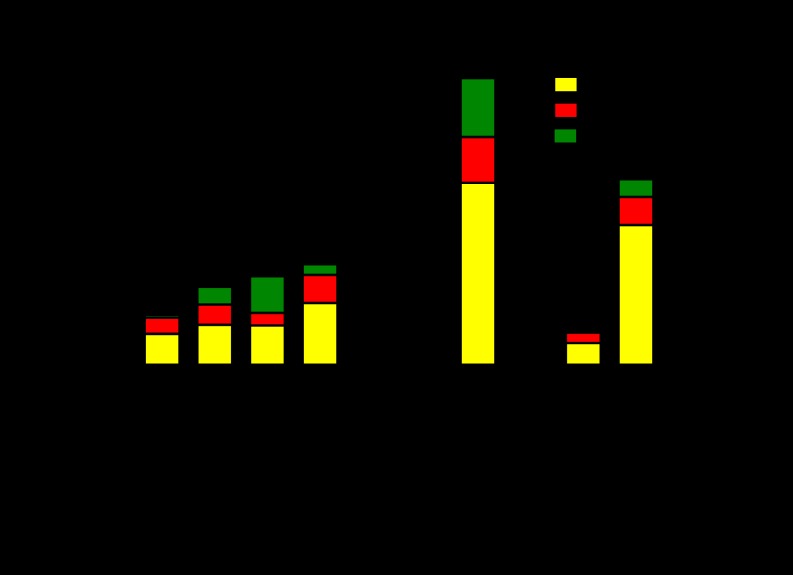
**Functional protein groups were made according to the classification of the TubercuList server, and for each functional group the identified proteins are presented as increased (>1.5-fold, *p* < 0.01, red), decreased (>1.5-fold, *p* < 0.01, green) or unchanged under starvation conditions (yellow).**

An overview of all identified proteins according to functional category is presented in Fig. 1, and the proteins are further divided to display increased, decreased, or unchanged abundance under starvation conditions. The majority of decreased proteins represent information pathways and intermediate metabolism and respiration, whereas the increased proteins are more broadly distributed in the functional categories of virulence, detoxification and adaptation, lipid metabolism, cell wall and cell processes, intermediate metabolism and respiration, and conserved hypotheticals.

The *M. tuberculosis* genome holds 168 predicted PE (Pro-Glu signature) and PPE (Pro-Pro-Glu signature) protein encoding genes (20), and strikingly, only one of these proteins (PE15, Rv1386) was identified in CF from log phase CF and starved bacteria (Fig. 1). A low extracellular abundance of these proteins believed to reside in the cell envelope might explain the lack of PE/PPE proteins identified in this study.

In order to further extract the biological interpretation of the LC-MS/MS dataset, a GO analysis utilizing the GoMiner algorithm was performed to look for overrepresentations of changed proteins in the associated GO categories. GoMiner was supplied with the list of 1176 identified proteins as the overall list and the list of the 438 changed proteins, including the direction of change. Of the 1176 identified proteins submitted as the overall list, 1051 had matches in GO, and the analysis then identified significant changes (*p* < 0.05) in GO categories between log phase CF and starvation CF (Table I).

##### GO Clustering Analysis of Starvation Decreased Proteins

Enriched GO terms in starvation CF decreased proteins comprise ribosomal proteins (Table I). This is in agreement with the down-regulation of ribosomal content and protein synthesis at the reduced growth rate under carbon starvation, which has also been observed in *Eschericia*, *Pseudomonas*, and *Vibrio* spp. (21–23). The presence of ribosomal proteins in CF is surprising at first sight; however, although the presence of a minor fraction of lysed cells cannot be ruled out, ribosomal proteins and other normally intracellular proteins such as elongation factor Tu and chaperonins (DnaK and GroEL) have also been detected in other Gram-positive bacteria, either on the surface of the bacteria or in the extracellular environment (24). In previous *M. tuberculosis* CF proteome studies, several ribosomal proteins were also detected (17, 25–27). The decrease of ribosomal proteins supports earlier findings at the gene level (3), and also in agreement with Betts *et al.*, we observed a significant decrease in proteins involved in the amino acid biosynthesis (Table I).

**Table I:** Representative enriched functional clusters for proteins identified by the GOMiner tool

GO category	Fold enrichment	Number of total proteins in category	Number of changed proteins in category	*p* value*^a^*	Proteins
Starvation-decreased proteins					
5840 Ribosome	4.49	48	41	<1.00 × 10^−4^	RplA, RplB, RplC, RplD, RplE, RplF, RplJ, RplM, RplN, RplO, RplP, RplQ, RplR, RplS, RplT, RplU, RplV, RplW, RplX, RplY, RpmA, RpmD, RpmE, RpmF, RpmJ, RpsB, RpsC, RpsD, RpsF, RpsG, RpsH, RpsI, RpsJ, RpsK, RpsM, RpsO, RpsP, RpsQ, RpsR1, RpsS, RpsT
51536 Iron–sulfur cluster binding	2.39	22	10	3.84 × 10^−3^	FdxA, GltD, IlvD, LeuC, NadA, NuoE, NuoF, NuoG, Rv2204c, Rv3818
8652 Cellular amino acid biosynthetic process	1.70	65	21	5.89 × 10^−3^	ArgB, ArgD, Asd, Cbs, DapB, DapF, FolD, GlnA2, GltB, GltD, HisC1, HisC2, IlvC, IlvD, IlvE, LeuB, LeuC, LeuD, Mec, RocA, SerC
Starvation-increased proteins					
16564 Transcription repressor activity	3.12	13	8	1.03 × 10^−3^	CmtR, Rv0144, Rv0158, Rv0328, Rv1219c, Rv1556, Rv3295, Rv3557c
40008 Regulation of growth	2.18	28	12	3.86 × 10^−3^	MazF6, ParE2, PknH, RelE2, VapB32, VapC13, VapC19, VapC22, VapC39, VapC4, VapC41, VapC5
15075 Ion transmembrane transporter activity	2.54	18	9	3.51 × 10^−3^	AtpA, AtpC, AtpD, AtpG, FecB, FecB2, ModA, PstS1, PstS2
4300 Enoyl-CoA hydratase activity	2.71	15	8	3.58 × 10^−3^	EchA1, EchA4, EchA5, EchA7, EchA8, EchA15, EchA16, EchA19
6779 Porphyrin biosynthetic process	2.90	7	4	3.11 × 10^−2^	CysG, HemC, HemZ, Rv1314c

^*a*^ The one-sided Fisher exact *p* value.

The group of iron–sulfur cluster binding proteins participate in a variety of cellular processes and was found to be significantly decreased in starvation CF (Table I). The identified decreased proteins with known functions served roles as ferredoxin (FdxA), in aerobic respiration (NuoE, NuoF, and NuoG), in amino acid synthesis (GltD, IlvD, and LeuC), and in the quinolinate synthetase (NadA) involved in the biosynthesis of NAD.

##### GO Clustering Analysis of Starvation Increased Proteins

Enriched clusters in starvation CF increased proteins included eight transcriptional repressors (Table I). Rv0144 has previously been predicted to be regulated by RelA, shown to be critical for establishing persistent infection in mice (28).

Enriched clusters in starvation CF increased proteins also included proteins involved in the regulation of growth as an overrepresented category of the increased proteins in the GoMiner analysis (Table I). The list of 12 increased proteins comprised the kinase PknH and 11 members of the toxin–antitoxin (TA) systems. The TA modules are numerous in the *M. tuberculosis* genome. Initially, 38 potential TA systems were described (29–31), but as many as 88 TA system candidates were later identified by Ramage *et al.* (32).

The enriched GO category ion transmembrane activity comprised nine starvation increased proteins, of which four form the ATP synthase enzyme complex (AtpA, C, D, and G), as well as lipoprotein members of the ABC transport systems including putative iron(III)-siderophore substrates (FecB and FecB2), the molybdate transport system (ModA), and phosphate uptake (PstS1 and PstS2), suggesting increased transport of iron, molybdate, and phosphate during the starvation conditions (Table I).

Enoyl-CoA hydratases were identified as significantly increased under starvation conditions (Table I). These enzymes serve a role in metabolizing fatty acids to produce acetyl CoA and energy, presumably reflecting increased β-oxidation of fatty acids in starvation cultures.

Four proteins involved in porphyrin biosynthesis were increased under starvation conditions (HemC, CysG, HemZ, and Rv1314c; Table I); Rv1314c is a probable Cob(I)alamin adenosyltransferase. The up-regulation of genes involved in the porphyrin biosynthesis pathway and transport was also observed under oxygen and carbon limitation (33).

Finally, the GoMiner analysis was also applied to look for changed proteins without considering the direction of change, and this analysis added lipid biosynthetic process (GO:0008610) as a significantly enriched term (*p* = 0.019; 34 altered proteins out of a total of 66 proteins were identified from this category (data not shown)).

Overall, the GoMiner clustering analysis reflected down-regulation of gene expression and growth, increased ion transport, and changes in lipid metabolism in the nutrient-starved cultures. Most of these observations support previous findings at the gene level under carbon starvation (3, 34). The specific findings from this study are described in more detail below.

##### Increased Abundance of TA Systems under Nutrient Starvation

The abundance of TA operons in *M. tuberculosis* and *M. bovis* is in contrast to other mycobacterial species such as *M. smegmatis*, *M. avium paratuberculosis*, and *M. leprae*, which contain few or no TA systems. This led to the hypothesis that *M. tuberculosis* could benefit from a regulation of growth in response to environmental stimuli mediated by TA systems, in contrast to *M. leprae*, which displays a less complex lifestyle within the host (29). TA systems typically consist of a two-gene operon encoding a downstream toxin and an unstable upstream antitoxin that inhibits the toxin. Further regulation is mediated by transcriptional autorepressor activity of the antitoxin, whereby proteolytic degradation of the antitoxin by stress-induced proteases leads to induction of the TA gene expression. The toxicity is based on either the blocking of DNA replication (DNA gyrase poison) or the inhibition of translation (ribonuclease activity). The *M. tuberculosis* genome encodes numerous proteases, but the protease(s) involved in antitoxin degradation has not yet been identified.

A significant increase in TA systems was detected by the GoMiner algorithm represented by 11 members of the TA systems, 2 antitoxins and 9 toxins. However, examination of the LC-MS/MS dataset identified four additional increased TA members that were not assigned the “regulation of growth” GO term; VapC27, VapC37, VapC38, and VapC44 (Table II). Eleven of the increased TA members represented the VapBC family of TA systems, which acts by inhibiting translation via mRNA cleavage, and nine of the identified TA systems (VapBC4, VapBC19, VapBC22, VapBC32, VapBC37, VapBC39, VapBC41, RelBE2, and MazEF6) were previously found to be functional (*i.e.* to limit growth) when toxin expression was induced in *M. smegmatis* (32, 35). Furthermore, three TA members identified in this study (VapBC4, MazEF6, and ParDE2) could limit growth in *Escherichia coli* (36). To our knowledge, this is the first confirmation at the protein level that the TA systems are induced during stress conditions possibly encountered during latent infection. Previous gene expression profiling data demonstrated induced gene expression of VapBC15 and HigAB1 during hypoxia and of VapBC3 and VapBC11 during infection by macrophages (32). Those TA members were not found to be increased in this study, suggesting distinct regulation of individual TA systems in response to different environmental stress conditions. Sequence-selective RNase activity has been demonstrated for VapC proteins, suggesting that the RNase specificity might vary as a result of the regulation of the VapCs (35). Furthermore, a recent study demonstrated examples of both physical and functional non-cognate TA interactions, indicating that crosstalk between *M. tuberculosis* TA systems might take place, which could further contribute to the fine regulation of the responses to environmental stress conditions (37).

**Table II:**
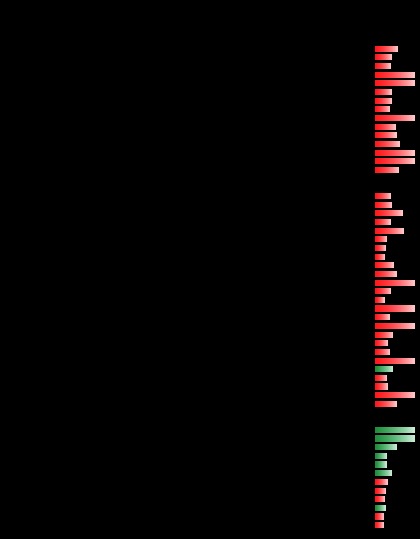
Proteins with changed abundance discussed in the text

^*a*^ Values in fold change represent the culture filtrate abundance ratio starvation/log phase.

^*b*^ The bars depict the log_10_ transformed fold change values; the red bars represent values between 0 and 2 (increased proteins), and the green bars represent values between 0 and −2 (decreased proteins).

The role of TA systems in bacterial stress physiology is a subject of much debate; one of the more widespread hypotheses is that TA systems induce growth arrest rather than cell death, resulting in the shutdown of protein synthesis until more favorable conditions occur (38).

##### Lipoproteins

Although not directly reflected in the GoMiner analysis, lipoproteins represent an enriched group of proteins under starvation, with 24 increased lipoproteins out of 62 identified in total (*p* = 0.0003); only one lipoprotein (Rv2934, PpsD) was identified with decreased abundance (Table II). Lipoproteins belong to the group of secreted proteins and are characterized by the presence of a lipobox immediately after the signal sequence. In addition to the previously described FecB, FecB2, ModA, PstS1, and PstS2, another three increased lipoproteins belonged to the ABC transport systems with a role in peptide transport (LpqZ, OppA, and DppA). The superoxide dismutase SodC plays a role in the antioxidant defense in *M. tuberculosis*. Other lipoproteins have predicted functions as glycosyl hydrolase (LpqI), lipoprotein aminopeptidases (LpqL and LpqM), and sensing and transmembrane signaling (LprA and LppH). The role of lipoproteins in immunopathogenesis has previously been emphasized for LpqT, LpqZ, and CaeA, which are all required for survival in primary murine macrophages (39) and were identified as increased under starvation in this study. GgtB is a gamma-glutamyl transferase involved in glutathione metabolism, and LpqW is involved in the synthesis of cell wall components. LprF is predicted to be in the RelA regulon (28) and was found to interact with the sensing domain of the histidine kinase KdpD (40). The function of the remaining lipoproteins with increased abundance (LppL, LppR, LpqB, LpqK, and LpqX) is unknown.

Bacterial lipoproteins have immunomodulatory activity and are capable of inducing IL-12 production mediated by Toll-like receptor 2 of host cells or the down-regulation of major histocompatibility complex expression and antigen processing (41). The latter mechanism and the observed inhibition of IFN-γ-induced responses could contribute to the ability of intracellular *M. tuberculosis* to maintain chronic infection and evade the immune system (42). Of the lipoproteins identified in this study, LprA and PstS1 have been shown to be inhibitors of MHC class II expression, and both are Toll-like receptor 2 agonists. Therefore, in addition to their roles as transport systems and in cell wall metabolism, it is possible that the increased extracellular abundance of lipoproteins during starvation can be explained by the demand for their immunomodulatory effects (43).

In the study by Betts *et al.*, lipoprotein-encoding genes were both down-regulated (10 genes) and up-regulated (7 genes) (3), and we speculate that the predominantly observed increase in CFs for the lipoproteins in our study could be due to the enhanced release of membrane vesicles, which have recently been characterized for *M. tuberculosis* (44). The vesicles can be recovered from culture supernatants, and they are enriched in lipoproteins. A role for these vesicles as carriers for immunomodulatory compounds like lipoproteins is therefore proposed for *M. tuberculosis*. In Gram-negative bacteria, the secretion of membrane vesicles has been thoroughly investigated, and it is characterized as a stress response with various functional roles including nutrient acquisition and toxin delivery to host cells (45).

##### T and B Cell Antigens

**Fig. 2.**
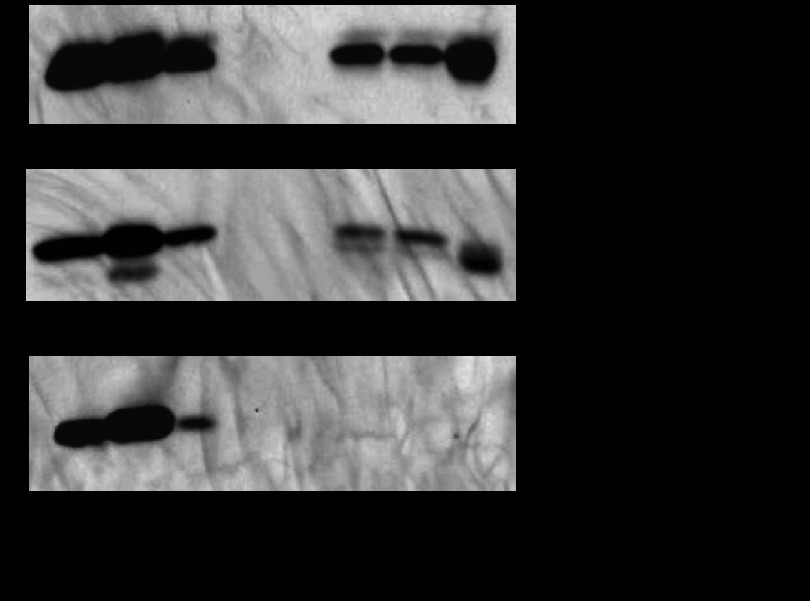
**Presence of Esx proteins in triplicate CF from log phase and 6-week-starved cultures.** Ten micrograms of each CF sample was tested via Western blot analysis with anti-EsxH/EsxR mouse monoclonal antibody, anti-EsxA mouse monoclonal antibody, and anti-EsxB rabbit polyclonal serum.

Antigenicity is not defined as a formal GO category, but we decided to look into this particular group of known T and B cell antigens, as the CF is considered as a source enriched for this group proteins. The ESAT-6 family comprises 23 members of ∼10-kDa proteins, and a large number of them have been identified as potent T cell antigens (46, 47). Six members of the secreted ESAT-6 family proteins were identified in this study (EsxA, EsxB, EsxG, EsxJ/EsxK, EsxL and EsxO), and all showed decreased abundance in starvation CF, although this decrease was not found to be significant for EsxG (0.09-fold, *p* = 0.039) according to the defined criteria. This suggests that expression of the *esx* genes is associated with the active growth of *M. tuberculosis* (Table II). The relationship between these proteins and their secretion by the type VII secretion system were not reflected by the associated GO terms. The *esxG* and *esxH* genes are co-transcribed (48), but neither EsxH (TB10.4) nor its homolog EsxR (TB10.3, 91% amino acid identity) were identified by LC-MS/MS in this study. The EsxH and EsxR amino acid sequences are 96 residues with trypsin cleavage sites at positions 67 and 86, and the resulting fragments can pose a challenge to confident mass spectrometry identification. By using a Mab specific for the linear epitope AGTLQSL in EsxH and EsxR,^2^ we confirmed via Western blot analysis the presence of EsxH/EsxR, EsxA, and EsxB in log phase CF, and this supports the decreased abundance of these proteins in CF from starved *M. tuberculosis* (Fig. 2).

Other previously described T cell antigens, PepA (49) and Apa (50), were also less abundant in starved CF. A recent study presented the unexpected finding that human T cell epitopes of *M. tuberculosis* are hyperconserved, possibly demonstrating that human T cell recognition is beneficial for the bacterium and perhaps required in order for *M. tuberculosis* to establish latent infection (51). The decreased abundance of some of these T cell antigens associated with the active growth of *M. tuberculosis* might indicate that once the bacterium has established a latent infection, it becomes advantageous for it to remain unrecognized by the immune system until conditions that induce resuscitation of the bacterium occur.

The FbpA (52) and the HBHA proteins, in contrast, were more abundant in starved CF, which for HBHA is in agreement with previous findings that the HBHA T cell antigen is a diagnostic marker for latent *M. tuberculosis* infection (53). Higher levels of the B cell antigens PstS1 and PstS2 (54) described above and Wag31 (55) were also found in starved cultures. Other interesting features of Wag31 in relation to the starvation conditions are its roles in the regulation of growth, cell wall synthesis, and cell morphology of mycobacteria (56).

##### Relative Protein Quantification via Two-dimensional DIGE and Label-free LC-MS/MS

**Fig. 3.**
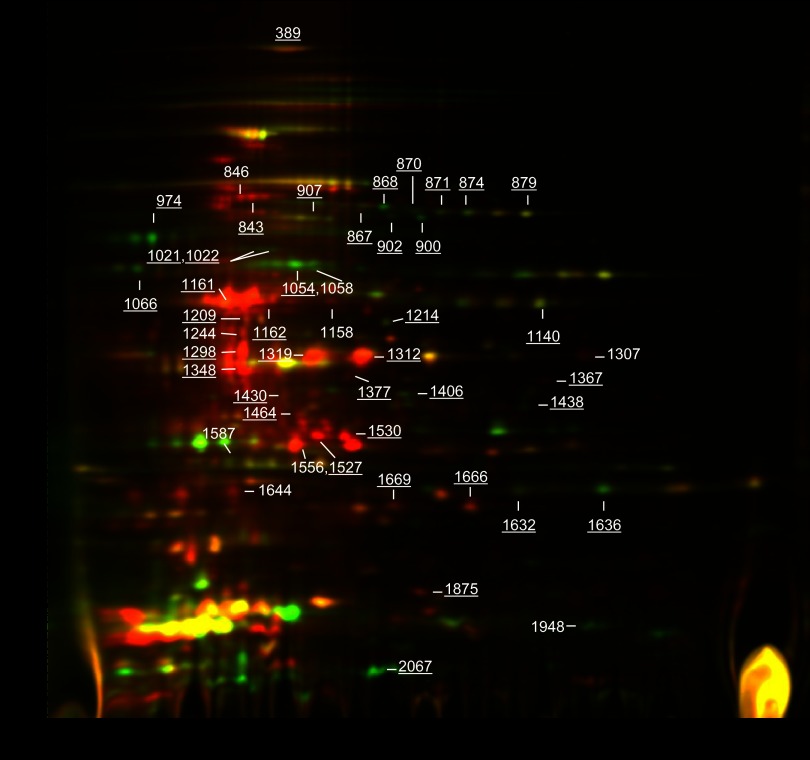
**Representative two-dimensional DIGE image of culture filtrate proteins from log phase and starvation conditions.** The total amount of protein used for CyDye labeling was 50 μg. The numbers on the left indicate molecular mass markers, and the numbers along the bottom of the image indicate the pI range. The numbered protein spots were excised from two-dimensional DIGE gels post-stained with silver and subjected to MS identification; for the underlined spot numbers, a successful identification was obtained, whereas no significant match was obtained in the Mascot search engine for spots not underlined. The spot numbering refers to the number of the spot assigned by the Imagemaster software. Protein spots that were more abundant in log phase appear as green, and spots more abundant under starvation conditions appear as red.

In parallel, the same set of CF samples collected from triplicates of log phase and starvation cultures were investigated via two-dimensional DIGE. The overall profile of both CF samples resembles that of previously published two-dimensional patterns for CF proteins (17, 25) (Fig. 3), and after the CF samples were stained with CyDyes, approximately 2100 spots were identified. The criteria for differentially abundant proteins were spots with average fold changes of a normalized volume ratio greater than 1.5 and a *p* value less than 0.05 (Student's *t* test). A total of 75 spots complied with the criteria for differential abundance. Of these, 48 spots were matched to the post-stained gel and were picked for MALDI-TOF-MS identification. Regarding the remaining 27 spots, the main reasons for not pursuing them were that unambiguous excision of the spots was not possible or the amount of protein in the spots was considered to be too low to allow identification by means of MS. MS analysis led to the positive identification of 39 spots, whereas for 9 spots no significant hit was obtained. The identified spots represented 26 unique proteins, reflecting that 9 proteins were identified in more than one spot. In one protein spot (#1464), two proteins (Rv1070c and Rv2716) were identified.

**Fig. 4.**
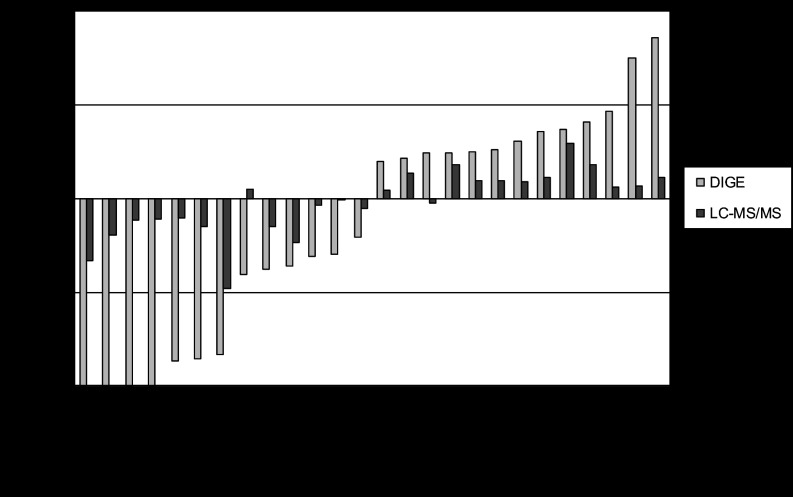
**Comparison of fold change observed with the two-dimensional DIGE method and the label-free LC-MS/MS approach.** Each bar shows the observed fold change in abundance (starved/log phase CF) as detected via the two-dimensional DIGE method (light gray bars representing the mean fold change of individual spots) and via the LC-MS/MS method (dark gray bars) for the individual proteins.

Overall, there was good agreement between the proteomic differences observed in two-dimensional DIGE and label-free LC-MS/MS (supplemental Table S4 and Fig. 4). Out of the 26 proteins detected via both techniques, there was complete agreement (based on both fold-change and statistical significance) for 15 proteins: 8 proteins were increased in two-dimensional DIGE (average spot volume ratio) and label-free LC-MS/MS (average spectral counts), and 7 proteins were decreased in the CF fraction from starved bacteria according to both methods. Furthermore, the seven proteins DnaK (Rv0350), Fba (Rv0363c), GroEL2 (Rv0440), FadA3 (Rv1074c), PrcA (Rv2109c), Rv2258c, and NdkA (Rv2445c) all showed the same direction of change with both methods, but these changes were not found to be statistically significant in the LC-MS/MS analysis. The remaining four proteins for which there was not agreement were LpdC (Rv0462), Fum (Rv1098c), CysK1 (Rv2334), and the hypothetical protein Rv2716. Rv2716 was identified as one of two proteins in spot 1464, and the quantitation in DIGE therefore refers to the mixture and not necessarily to both individual proteins.

Two of the apparent inconsistencies between the two methods can be explained by the capability of two-dimensional DIGE to detect different post-translationally modified variants or species of the same proteins, whereas the LC-MS/MS experiment quantifies the average of all present species of a protein. For example, a total of five distinct two-dimensional DIGE spots were identified as LpdC. Four of the LpdC spots were significantly decreased and one was increased in starved CF. According to the label-free LC-MS/MS, however, there was no significant difference in the spectral counts of LpdC between the CF from log phase and starved cultures. In the two-dimensional DIGE analysis, the four decreased LpdC spots (spots 870, 871, 874, and 879) were part of a horizontal charge-train of ∼50 kDa (Fig. 3), and this is in good agreement with the theoretical mass of LpdC of 49.2 kDa. The structural differences between these four LpdC spots are unknown. The last LdpC spot (1367) was observed at ∼20 kDa, and, conversely, this spot was increased in starved CF. In addition, a total of four two-dimensional DIGE spots were identified as GroEL2 (spots 1348, 1430, 1527, and 1530), and the abundances of all four spots were significantly increased after starvation. In the LC-MS/MS analysis, GroEL2 was also increased after starvation but did not quite meet our inclusion criteria for increased spots (1.49-fold, *p* = 0.0047). The theoretical mass of GroEL2 is 57 kDa, and all four GroEL2 spots were present in the range from ∼35 to 15 kDa (Fig. 3). The spot patterns of LdpC and GroEL2 suggest increased proteolytic activity in starvation cultures. For GroEL2, Western blot with monoclonal anti-GroEL2 further supported proteolytic degradation in 6-week starvation CF when compared with the band representing the intact protein in the log phase CF (supplemental Fig. S2).

Nine of the proteins identified with altered abundance via two-dimensional DIGE were present in two or more spots representing different species of the protein, which illustrates the capability of the two-dimensional approaches to separate different protein species and allow the characterization of potential protein-species-specific regulation (57). For eight of the proteins, the direction of change was identical for all individual spots, whereas for LpdC both increased and decreased abundance was observed for individual spots (supplemental Table S4). This illustrates that ideally, all spots in the two-dimensional DIGE gels should be identified in order to uncover the presence of additional protein species that might be differently regulated.

## CONCLUDING REMARKS

In this study we completed a comprehensive comparison of the proteome profiles of *M. tuberculosis* CF from log phase and starvation via two different proteomics approaches. The results allowed us to gain biological insight into the processes occurring when *M. tuberculosis* is adapting to conditions that mimic latent disease. The GO clustering analysis proved to be a valuable tool for interpreting large proteomic datasets, and intriguing new findings in our study were the numerous members of the TA systems identified as increased under conditions that mimic latency. TA modules are highly debated, and intense efforts are currently being made to elucidate their mechanism as “stress managers” in mycobacteria, as well as in other bacteria. In CF from starved bacteria, the abundance of lipoproteins was also increased (several of them are involved in nutrient transport systems), whereas several known T cell antigens, including the ESAT-6 family proteins, showed decreased abundance.

We confirmed several findings from the previously published microarray studies of carbon limitation (3, 34), although differences were also observed, as, for example, described for the lipoproteins, where our focus on the subset of proteins released to the extracellular environment could give rise to a different protein profile. Other observed differences between this study and the previous microarray studies can be ascribed to overall differences in measuring protein and mRNA levels, the turnover of the individual proteins, and regulation via the post-translational modification of proteins, as observed for LpdC, which is detectable only by means of various proteomics techniques. Although the GoMiner analysis proved to be an important tool in the data analysis, it was also noted that the GO annotation was not always complete and consistent, and the automated analysis required manual evaluation as pointed out earlier for microbial proteomes (58).

To our knowledge, we have provided the first comparison of two-dimensional DIGE *versus* label-free LC-MS/MS. We emphasize that disagreements in protein abundance as determined by means of two-dimensional DIGE and LC-MS/MS can also be ascribed to subtler analytical/technical aspects of either technique. Overall, however, the presence of few inconsistencies in the present study supports the idea that both two-dimensional DIGE and label-free LC-MS/MS are reliable approaches for the relative protein quantification of complex protein mixtures. It was also evident from the comparison that the sensitivity obtained with the LC-MS/MS technique was higher, although further optimization of the two-dimensional DIGE system by, for example, the use of larger gels and non-equilibrium pH gel electrophoresis carrier ampholytes (59), prefractionation of the samples, or the use of narrow pH-range immobilized pH gradient strips could lead to improved sensitivity.

The extracellular proteins of *M. tuberculosis* might interact directly with the host, and therefore they constitute an important source of potential vaccine and therapeutic targets. The distinct proteome profile of nutrient-starved bacteria confirms that other targets should be considered for combating latent infection and emphasizes that the potential of many “new” proteins, such as the TA systems, remains to be explored. In future work on the characterization of TA systems, it will be important to identify the protease(s) involved in antitoxin degradation and the cellular targets of the individual toxins in *M. tuberculosis*. This will hopefully lead to more insights into the mechanism of these unusually abundant systems in this pathogen.

## Supplementary Material

Supplemental Table 1

## Footnotes

^2^ I. Rosenkrands, unpublished data.

^1^ The abbreviations used are:

CF: culture filtrate
cfu: colony-forming unit
DIGE: difference gel electrophoresis
ESAT-6: early secretory antigen target, 6 kDa
GO: gene ontology
Mab: monoclonal antibody
TA: toxin–antitoxin.
