# Supplementary material for: Proteomic Profiling of Mycobacterium tuberculosis Identifies Nutrient-starvation-responsive Toxin–antitoxin Systems
Source: Mol Cell Proteomics. 2013 Jan 23;12(5):1180–91. doi: 10.1074/mcp.M112.018846 (PMC3650330; doi:10.1074/mcp.M112.018846)
Supplement: Supplemental Table 1 [file supp_12_5_1180__index.html]

Proteomic profiling of the Mycobacterium tuberculosis identifies nutrient starvation responsive toxin-antitoxin systems — Proteomic Profiling of Mycobacterium tuberculosis Identifies Nutrient-starvation-responsive Toxin–antitoxin Systems — M. tuberculosis Nutrient-starvation-responsive Proteins — Supplemental Table 1 

# Proteomic Profiling of *Mycobacterium tuberculosis* Identifies Nutrient-starvation-responsive Toxin–antitoxin Systems

## Supplemental Data

**Files in this Data Supplement:**

- Supplemental Table 4 - Comparison of hits identified by 2D DIGE with LC-MS/MS results.
- Supplemental Figure 1 - SDS-PAGE profile of log phase and six-week starved CF samples.
- Supplemental Figure 2 - Presence of GroEL2 protein in triplicate CF from log phase and six-week starved cultures.
- Supplemental Table 1 - Peptide and protein identifications from LC-MS/MS analysis of triplicate samples from log phase and starvation CF.
- Supplemental Table 2 - Spectral counts from LC-MS/MS analysis of triplicate samples from log phase and starvation CF. Proteins identified in all three log phase CF samples and in all three starvation CF samples are listed as separate tables. A merged list of the proteins identified in log phase versus starvation CF is also shown.
- Supplemental Table 3 - Proteins with different abundance in starvation versus log phase CF identified by LC-MS/MS.
