# Supplementary material for: Proteomic Profiling of Mycobacterium tuberculosis Identifies Nutrient-starvation-responsive Toxin–antitoxin Systems
Source: Mol Cell Proteomics. 2013 Jan 23;12(5):1180–91. doi: 10.1074/mcp.M112.018846 (PMC3650330; doi:10.1074/mcp.M112.018846)
Supplement: Supplemental Table 1 [file supp_M112.018846_mcp.M112.018846-4.pdf]

Suppl table 4. Comparison of hits identified by 2D DIGE with LC-MS/MS results

2D DIGE												LC-MS/MS		2D DIGE vs. LC-MS/MS		
Match ID	Gene	Function	% Coverage	Mascot score	Method	Matched	Unmatched PMF	Theor. Mass	Theor. pI	p value	Average fold change	p value	Average fold change	DIGE[#]	LC-MS/MS[#]	Correspondance
1632	Rv0009	PpiA, peptidyl-prolyl cis-trans isomerase A	57	66	PMF	9	79	19.2	6.23	1.40E-02	0.01	5.9E-04	0.62	🟢	🟢	Yes
1636	Rv0009	PpiA, peptidyl-prolyl cis-trans isomerase A	57	59	PMF	9	100	19.2	6.23	2.10E-02	0.12			🟢		
907	Rv0234c	GabD1, succinate-semialdehyde dehydrogenase	59	188	PMF	17	26	54.3	5.22	1.40E-02	0.01	6.0E-04	0.22	🟢	🟢	Yes
1298	Rv0350	DnaK (hsp70), 70 kDa heat shock protein	22	130	PMF	12	15	66.8	4.59	4.60E-04	8.54	9.0E-04	1.34	🔴	🟡	No
1140	Rv0363c	Fba, fructose biphosphate aldolase	20	380	MS/MS	4		38.7	6.18	3.00E-02	0.39	6.5E-03	0.78	🟢	🟡	No
843	Rv0418	LpqL, lipoprotein peptidase	52	163	PMF	15	29	52.0	4.78	3.90E-02	5.52	7.7E-05	3.84	🔴	🔴	Yes
1348	Rv0440	GroEL2, 60 kDa chaperonin 2	29	125	PMF	11	14	56.7	4.56	2.70E-04	9.96	2.5E-02	1.37	🔴	🟡	No
1430	Rv0440	GroEL2, 60 kDa chaperonin 2	40	87	PMF	14	85	56.7	4.56	1.00E-02	3.59			🔴		
1527	Rv0440	GroEL2, 60 kDa chaperonin 2	31	74	PMF	13	54	56.7	4.56	1.20E-02	100			🔴		
1530	Rv0440	GroEL2, 60 kDa chaperonin 2	24	110	PMF	10	11	56.7	4.56	4.20E-02	13.39			🔴		
870	Rv0462	LpdC, dihydrolipoamide dehydrogenase	8	86	MS/MS	2		49.2	5.70	2.80E-02	0.10	9.4E-01	0.99	🟢	🟡	No
871	Rv0462	LpdC, dihydrolipoamide dehydrogenase	59	200	PMF	20	33	49.2	5.70	3.10E-02	0.14			🟢		
874	Rv0462	LpdC, dihydrolipoamide dehydrogenase	68	201	PMF	24	52	49.2	5.70	2.70E-03	0.17			🟢		
879	Rv0462	LpdC, dihydrolipoamide dehydrogenase	4	77	MS/MS	1		49.2	5.70	4.30E-02	0.31			🟢		
1367	Rv0462	LpdC, dihydrolipoamide dehydrogenase	35	86	MS/MS	12		49.2	5.70	6.40E-03	7.16			🔴		
1377	Rv0831c	Conserved hypothetical protein	56	202	MS/MS	16		30.2	4.85	7.50E-04	6.50	4.1E-04	2.30	🔴	🔴	Yes
1021	Rv0884c	SerC, phosphoserine aminotransferase	66	172	PMF	17	35	40.2	4.53	3.70E-03	0.01	1.7E-04	0.41	🟢	🟢	Yes
1022	Rv0884c	SerC, phosphoserine aminotransferase	48	105	PMF	15	84	40.2	4.53	1.80E-03	0.01			🟢		
1162	Rv0934	PstS1, phosphate-binding lipoprotein	39	56	PMF	7	42	38.2	5.02	4.70E-02	2.71	1.9E-03	1.86	🔴	🔴	Yes
1054	Rv1074c	FadA3, beta-ketoacyl CoA thiolase	56	205	PMF	20	20	42.7	4.67	6.70E-03	0.01	2.9E-02	0.60	🟢	🟡	No
867	Rv1077	Cbs, cysteine beta synthase	48	131	PMF	17	74	48.6	4.94	4.50E-02	0.33	6.9E-03	0.51	🟢	🟢	Yes
868	Rv1077	Cbs, cysteine beta synthase	51	199	MS/MS	24		48.6	4.94	7.30E-03	0.01			🟢		
900	Rv1098c	Fum, fumarate hydratase	33	139	PMF	12	17	50.1	5.20	3.50E-05	0.13	2.1E-01	1.27	🟢	🟡	No
902	Rv1098c	Fum, fumarate hydratase	33	102	PMF	12	40	50.1	5.20	1.40E-04	0.18			🟢		
1666	Rv1284	CanA, Beta-carbonic anhydrase	71	89	PMF	12	84	18.2	5.59	1.20E-03	100	3.4E-03	1.70	🔴	🔴	Yes
1669	Rv1284	CanA, Beta-carbonic anhydrase	52	566	MS/MS	6		18.2	5.59	4.10E-02	2.62			🔴		
389	Rv1475c	Acn, aconitate hydratase	9	88	MS/MS	5		102.4	4.70	9.50E-04	3.14	1.9E-03	1.53	🔴	🔴	Yes
974	Rv1860	Apa, Ala-, Pro-rich 45/47 kDa secreted protein	27	225	MS/MS	7		32.7	4.7	3.50E-02	0.11	3.4E-03	0.50	🟢	🟢	Yes
1066	Rv1860	Apa, Ala-, Pro-rich 45/47 kDa secreted protein	20	236	MS/MS	6		32.7	4.7	4.50E-03	0.41			🟢		
1406	Rv2109c	PrcA, proteasome alpha-type subunit 1	68	217	PMF	18	36	28.3	4.52	6.60E-03	0.01	4.0E-02	0.59	🟢	🟡	No
1209	Rv2145c	Wag31 conserved hypothetical protein	41	164	PMF	10	5	28.3	4.52	2.90E-02	3.28	2.4E-03	1.56	🔴	🔴	Yes
1161	Rv2258c	Possible transcriptional regulatory protein	65	144	PMF	21	55	37.5	4.35	2.30E-02	2.49	5.8E-02	1.23	🔴	🟡	No
1214	Rv2334	CysK1, cysteine synthase A	20	376	MS/MS	5		32.8	4.93	6.40E-03	0.24	1.8E-01	0.84	🟢	🟡	No
1875	Rv2445c	NdkA, nucleoside diphosphate kinase	62	98	PMF	9	61	14.5	5.18	2.80E-02	5.18	3.2E-02	1.67	🔴	🟡	No
1438	Rv2773c	DapB, dihydrodipicolinate reductase	46	122	PMF	11	39	25.7	5.75	6.70E-04	0.19	2.8E-04	0.34	🟢	🟢	Yes
2067	Rv3648c	CspA, cold shock protein A	65	226	MS/MS	2		7.4	4.06	5.40E-04	0.02	4.0E-03	0.11	🟢	🟢	Yes
1312	Rv3804c	FbpA, mycolyl transferase 85A	13	146	MS/MS	2		35.7	6.51	3.00E-03	4.97	6.3E-03	1.53	🔴	🔴	Yes
1319	Rv3804c	FbpA, mycolyl transferase 85A	13	182	MS/MS	3		35.7	6.51	1.40E-02	3.15		1.53	🔴		
1464	Rv2716	Conserved hypothetical protein	56	85	PMF	9	48	24.6	4.75	3.80E-03	3.08	4.9E-01	0.89	🔴	🟡	No
	Rv1070c	EchA8, enoyl-CoA hydratase	42	90	PMF	12	48	27.3	4.67	3.80E-03	3.08	4.0E-04	2.27	🔴	🔴	Yes
846	Unidentified	-	-	-	PMF	-		-	-	3.00E-02	5.18	-	-	-	-	-
1058	Unidentified	-	-	-	PMF	-		-	-	1.40E-02	0.24	-	-	-	-	-
1158	Unidentified	-	-	-	PMF	-		-	-	4.20E-03	2.59	-	-	-	-	-
1244	Unidentified	-	-	-	PMF	-		-	-	3.89E-04	100	-	-	-	-	-
1307	Unidentified	-	-	-	MS/MS	-		-	-	2.74E-06	8.64	-	-	-	-	-
1556	Unidentified	-	-	-	MS/MS	-		-	-	2.40E-02	5.56	-	-	-	-	-
1587	Unidentified	-	-	-	MS/MS	-		-	-	2.20E-02	4.08	-	-	-	-	-
1644	Unidentified	-	-	-	MS/MS	-		-	-	2.40E-03	100	-	-	-	-	-
1948	Unidentified	-	-	-	MS/MS	-		-	-	1.10E-02	8.33	-	-	-	-	-

#The following symbols are used: Green circles means decreased abundance in starvation CF, Red circles means increased abundance in starvation CF and and yellow circles no significant change in starvation CF
