# Supplementary material for: Proteomic Profiling of Mycobacterium tuberculosis Identifies Nutrient-starvation-responsive Toxin–antitoxin Systems
Source: Mol Cell Proteomics. 2013 Jan 23;12(5):1180–91. doi: 10.1074/mcp.M112.018846 (PMC3650330; doi:10.1074/mcp.M112.018846)
Supplement: Supplemental Table 1 [file supp_M112.018846_mcp.M112.018846-5.pdf]

Albrethsen et al, Supplemental figure 1

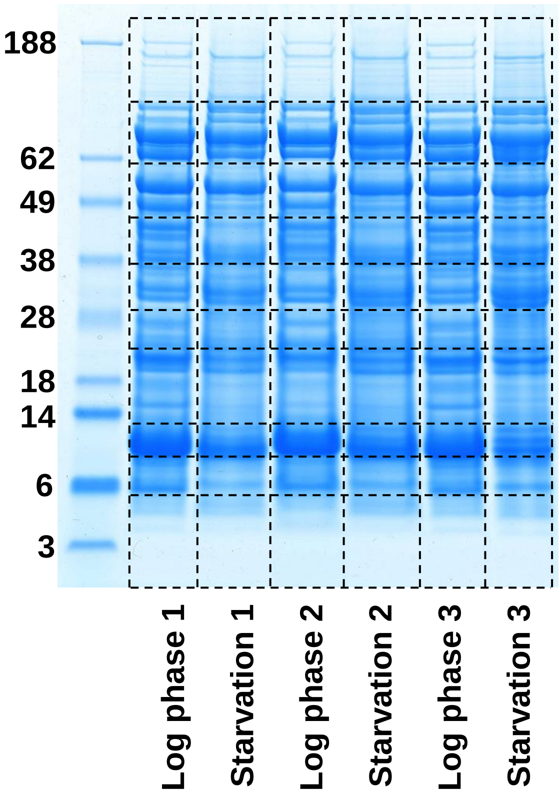

Suppl. Fig. 1. SDS-PAGE gel of log phase and starvation CF samples for LC-MS/MS analysis. Sixty µg of each CF sample was applied on the gel followed by Coomassie staining and excision of 10 individual bands from each lane as indicated. Molecular mass markers are shown.
