# Supplementary material for: Proteomic Profiling of Mycobacterium tuberculosis Identifies Nutrient-starvation-responsive Toxin–antitoxin Systems
Source: Mol Cell Proteomics. 2013 Jan 23;12(5):1180–91. doi: 10.1074/mcp.M112.018846 (PMC3650330; doi:10.1074/mcp.M112.018846)
Supplement: Supplemental Table 1 [file supp_M112.018846_mcp.M112.018846-6.pdf]

Albrethsen et al, Supplemental figure 2

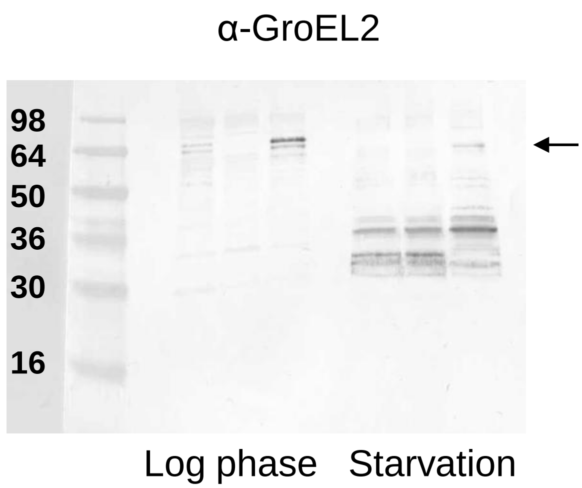

Suppl. Fig. 2. Presence of GroEL2 protein in triplicate CF from log phase and six-week starved cultures. Ten μg of each CF sample was tested by Western blot analysis with anti-GroEL2 mouse monoclonal antibody. Molecular mass markers are indicated. The position of the intact protein is marked with an arrow.
